# Experimental Study on Deep-Drawing Dies Made of Pre-Stressed UHPC

**DOI:** 10.3390/ma18020277

**Published:** 2025-01-09

**Authors:** Katja Holzer, Yuqi Zhang, Lukas Martinitz, Julika Hoyer, Wolfram Volk

**Affiliations:** Chair of Metal Forming and Casting, Technical University of Munich, Walther-Meissner-Strasse 4, 85748 Garching, Germanylukas.martinitz@tum.de (L.M.); julika.hoyer@tum.de (J.H.); wolfram.volk@tum.de (W.V.)

**Keywords:** ultra-high-performance concrete, expansive concrete, restrained condition, prototype tooling, deep drawing

## Abstract

Deep drawing is a cost-efficient way of producing sheet metal parts in high production volumes. Prototypes and very small series are expensive due to the cost of steel-forming tools. Ultra-high-performance concrete (UHPC) tools offer a cheap and fast alternative to conventional steel-forming tools. However, the flexural and tensile strength of UHPC limits its use in complex loading situations occurring in the forming die during deep drawing. To overcome this challenge, we propose pre-stressing UHPC by using expansive UHPC in a mechanically restrained condition. An aluminum powder-based expansive agent can be used to induce a free volume increase in UHPC specimens. With push-out test specimens, the increase in strength was tested when restraining the free volume increase with a steel reinforcement. After 3 and 12 months, no notable pre-straining effect could be measured when restraining the expansion of UHPC. Nonetheless, in deep-drawing experiments, the die made of expansive UHPC in a restrained condition withstood a maximal load during deep drawing of 110 kN. Compared to the die made from UHPC only, the failure mode changed from complete fracture to surface degradation of the drawing radius after 15 strokes.

## 1. Introduction

Deep drawing is a cost-efficient way of producing sheet metal parts in high production volumes. Small numbers of deep-drawn parts are expensive due to the cost of steel-forming tools. An approach to reducing the production costs of prototypes, spare parts, and individual parts is using flexible tooling made of plastic materials. Forming tools can be made from milled polymers or casted resins [1,2]. With the ongoing advancements of additive technologies, directly printed polymeric tools become feasible. Direct polymer additive tooling can be produced by fused filament fabrication (FFF) with polylactic acid (PLA) [3,4,5], polyamide (PA) [6], and CF-reinforced PA [7]. The tolerances are rarely met when drawing more complex geometries with direct polymer additive tooling [8,9]. The dimensional accuracy of the drawn part is directly influenced by the low stiffness of the polymeric tools [10].

Ultra-high-performance concrete (UHPC) tools offer a cheap and fast alternative to conventional steel-forming tools with higher stiffness [11]. UHPC has a higher packing density compared to conventional concrete due to the small particle size of the binder and the use of additives. This accelerates the hydration reaction and gives higher strength properties [12]. UHPC was already combined with a gel coat to produce sheet metal parts for prototype cars [13]. UHPC dies were also successfully used for the hydroforming of sheet metal [14,15,16]. One of the challenges in the use of UHPC tools for deep drawing is the complex stress state induced by the tools.

To increase the strength of concrete parts, pre-stressing can be used. Conventional pre-stressing of concrete and UHPC is performed by externally applying tensile stress on the tendons. This can be performed before casting or after curing of the concrete. In both ways, equipment for tensioning the tendons is needed as well as a frame to anchor the tendons. The concrete becomes pre-stressed by releasing the anchoring of the tendons, transferring compression from the tendons through the bond into the concrete [17]. Conventional pre-stressing is limited by the direction in which pre-stress can be applied. In known applications, pre-stress is applied in one direction only. For forming tools, this is not acceptable, as the main stress direction during deep drawing depends on the shape that is deep drawn, i.e., strongly alternating in the case of industrial components.

Another method of introducing compressive stresses into the concrete part is chemical pre-stressing. Here, an expansive agent is added to the concrete mixture. This leads to an increase in volume that, if properly restrained, will induce compressive stresses in the concrete by limiting expansion with a reinforcement [18,19]. Chemically pre-stressed concrete (CPC) or expansive concrete in a restrained condition is used for producing concrete pipes [20] and thin-walled structures [21] and can be used with further reinforcement like CFRP tendons [22]. With the right admixtures, expansive agents can increase the durability and mechanical properties of high-performance concrete [23]. The expansive agents used consist of iron powder, aluminum powder, magnesia, calcium sulfoaluminate, or calcium oxides [24]. Calcium sulfoaluminate is the most commonly used.

The addition of aluminum powder to concrete causes a chemical reaction that produces hydrogen gas [25]. As the gas develops, the air content in the concrete rises and expands the part. With an increasing amount of aluminum, the expansion rate and the final expansion of the concrete part increase [25,26]. Aluminum powder, e.g., powder made of produce waste, is mostly used as a cost-effective alternative to autoclaved aerated concrete for the production of foamed concrete and ultra-lightweight concrete structures [27,28,29]. Compared to other expansive agents, aluminum powder does not form a compound that causes the concrete to expand, like ettringite formation by adding calcium sulfoaluminate [30]. The concrete strength reduces with an increased amount of aluminum added. When restraining the volume expansion, the strength can be restored according to the final volume achieved by the fresh mixture [26].

In this work, we propose the use of an aluminum powder-based expansive agent to pre-stress UHPC by restraining deep-drawing tools. First, we investigate the increase in free volume introduced by two different commercially available expanding agents in UHPC specimens. Therefore, the density was determined using Archimedes’ principle. We try to clarify if an expansion in volume (a decrease in density) of UHPC specimens is possible with an aluminum powder-based expansive agent. Additionally, we investigate if the expansive behavior induced in the UHPC of two commercially available expansive agents is equal if both are based on aluminum powder. Secondly, in push-out test specimens, we utilize the expansion of the UHPC to pre-stress by using a steel ring as reinforcement. We create specimens as well as a setup to test expansive UHPC in a mechanically restrained condition. Expansive agents are added to the UHPC in varying quantities and filled into an outer steel ring before curing. The specimens are loaded onto the UHPC matrix in axial direction until fracture. This test answers the question of whether it is possible to pre-stress the specimens using an outer steel reinforcement in order to mechanically restrain the expansion of UHPC caused by an aluminum powder-based expansive agent. Setting a quantity of expansive agents, deep-drawing experiments were performed as proof of concept. We compare a die made of UHPC with one made of expansive UHPC in a restrained condition. A cup drawing tool with an open die is used for the experiments. With the deep-drawing experiments, we want to clarify if reinforcing a deep-drawing die with an inner and outer steel ring and expansive UHPC in a restrained condition improves performance compared to a UHPC die without reinforcement.

## 2. Materials

### 2.1. UHPC Composition and Admixtures

Two expansive agents are considered in this study. Both are commercially available products in powder form used for the production of swelling concretes and swelling mortars. Both agents correspond to the standard DIN EN 934-4 for concrete admixtures [31]. The active ingredient in both expansive agents is aluminum powder, which is not further specified by the manufacturers. The agents are as follows: expansive agent 1—DARAGROUT Expand (ST) by GCP Applied Technologies, Cambridge, MA, USA [32], and expansive agent 2—Premadd Stabilisator QM 25 by BT3 Betontechnik GmbH, Theresienfeld, Austria [33]. These two expansive agents were selected because they are easily accessible and inexpensive to procure. This is particularly important with regard to the quantity required for large UHPC-forming tools. In addition, according to the manufacturers, the active ingredient of both expansive agents is the same. However, it cannot be concluded that they have the same properties.

All test specimens were made based on the base composition of UHPC given in Table 1. As a binder, we choose the ready-to-use compound Nanodur Compound 5941 from Dyckerhoff GmbH, Wiesbaden, Germany. In addition to cement and synthetic silicic acid, this compound contains quartz powder and can be used to produce UHPC without further additives [34]. To prevent the formation of agglomerates due to the steric effect and to improve the workability of the fresh concrete, a superplasticizer is added to the mixing water [18]. Here, the additive ADVA Flow375 (BV/FM) by GCP Applied Technologies, Cambridge, MA, USA, is used with an active ingredient based on polycarboxylate ethers (PCE) [35]. PCE further helps to ensure a homogeneous and pore-free microstructure of the final product [12,36]. The composition is easy to process and is self-compacting. No further compaction process for de-aeration was used. In compression tests with a 30 mm × 30 mm cubic specimen made of the base composition, a compressive strength of 200 MPa was achieved. The flexural strength of the 30 mm × 30 mm × 160 mm specimen in the three-point bending test was tested with a span of 100 mm. The flexural strength was found to be 17 MPa.

To the base composition of UHPC, we added the expansive agent in weight percent of the binder weight. The additions are varied from 0.2 to 2 wt.% as advised in the datasheets. The corresponding water–binder ratios (w/b) of the specimen are given in Table 2, whereas the amount of superplasticizer was added to the mass of water.

For the free volume increase test, specimens with 0.2, 0.5, 1, 1.5, and 2 wt.% of expansive agents 1 and 2, respectively, were produced.

Test specimens for the push-out test were made based on the base composition of UHPC with the addition of 0.2 to 2 wt.% of expansive agent 2. For expansive agent 1, only specimens with 1 wt.% were produced.

The composition of UHPC used for the deep-drawing experiments is given in Table 3. For the die made from UHPC, the base composition is used (Table 1), and 2 wt.% of expansive agent 2 is added to fabricate the die made from expansive UHPC in a restrained condition.

### 2.2. PLA Molds

All molds used in this work to cast UHPC specimens are made of PLA fabricated by fused filament fabrication. The filament used is made of PLA with a diameter of 1.75 mm purchased by Das Filament, Emskirchen, Germany. These molds can only be used once and are destroyed when removing the UHPC cast from the mold.

For the push-out test specimen, a PLA mold with an inserted outer steel ring was used. The steel ring was inserted in the bottom mold, filled with UHPC mixture, and pressured for the duration of curing on the upper mold. The steel ring, as well as the applied pressure, ensures the pre-stressing of the specimen by mechanically restraining the expansion of the UHPC composition. The outer steel ring has a thickness of 2 mm and an outer diameter of 42 mm. The steel rings were cut out of a welded tube of stainless steel and ground. The height varies between 9.94 and 12.2 mm.

**Figure 1:**
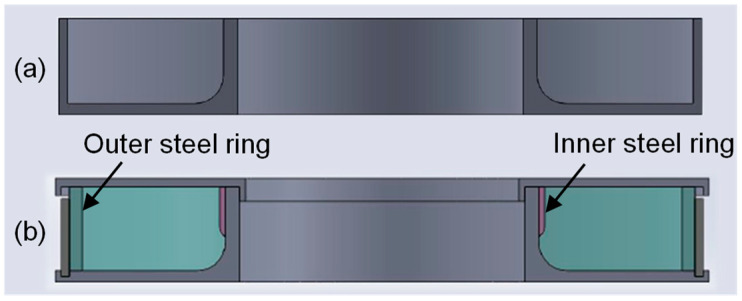
Schematic of the molds fabricated by fused filament fabrication for (**a**) reference die made of UHPC and (**b**) die made of expansive UHPC in a restrained condition with outer and inner steel ring and cover.

The PLA molds used for casting the deep-drawing dies are shown in Figure 1. The mold for the reference die made of UHPC is shown in Figure 1a. In the mold for the die made of expansive UHPC, inner and outer steel rings are inserted before casting. The steel rings were cut out of a welded tube made of unalloyed structural steel. The outer steel rings were cut to a height of 30 mm and ground down. The inner steel ring was cut to a height of 20 mm to leave room for the drawing radius of 10 mm. The inside of the inner ring was chambered to allow for better adhesion of the UHPC and better force transmission. This mold also has a cover to pressurize the expansive UHPC during curing. The mold for the pre-stressed die is shown in Figure 1b.

### 2.3. Specimen Preparation and Curing

The constituents (binder, water, superplasticizer, and expansive agent) were weighted with an accuracy of ±0.1 g and mixed by hand for at least 12 min. The mixture was mixed until a homogeneous consistency with dilatant behavior was obtained and poured into the mold.

For the free volume test specimens, a 300 g mixture for each configuration was prepared and divided into three specimens. The specimens were cured for 24 h in paper cups in a saturated atmosphere at room temperature. After 24 h, half of the demolded specimens were tested right away. The other half was stored dry at room temperature for 7 days before testing.

**Figure 2:**
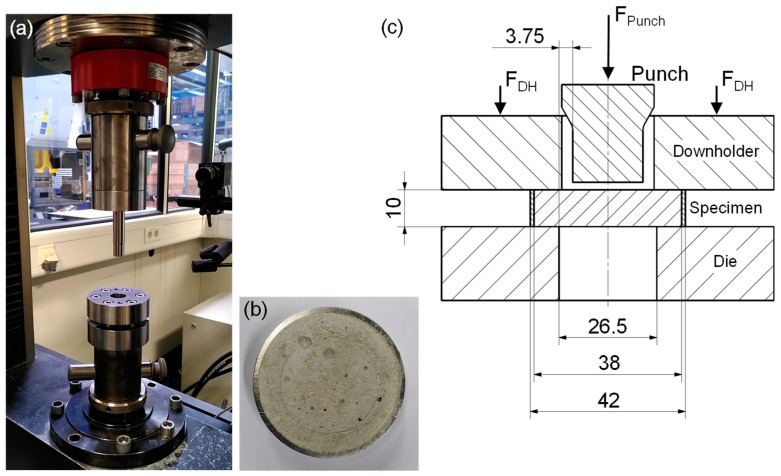
(**a**) Push-out test setup mounted in the universal testing machine, (**b**) push-out test specimen after fracture, and (**c**) schematic of the push-out test setup with notable dimensions in mm.

For the push-out test specimens, a 150 g mixture for each configuration was prepared. Inside one casting, 50 g of UHPC mixture was poured. Three specimens were produced from one mixture. Curing was performed inside the pressured mold in saturated atmosphere at room temperature for 24 h. After demolding, the specimens were ground on both sides with a rotating disk grinder. This is necessary to obtain a flat contact surface and to avoid stress peaks in the contact surface. Afterward, the specimens were stored in water at room temperature for 3 and 12 months before testing. The specimens were tested after this time to account for time used for fabrication and reworking of possible UHPC-forming tools (3 months) and to investigate storage effects (>12 months). Figure 2b shows a push-out test specimen after fracture.

For each deep-drawing die, 850 cm^3^ of the respective UHPC composition given in Table 3 was prepared. The dies were cured for 24 h in saturated atmosphere. After demolding, the only rework performed was surface grinding to fabricate an even surface for mounting both dies. The dies were stored dry for 7 days until testing. Keeping the dies dry is important as they can only be used dry in deep-drawing applications. Any moisture has to be fully prevented to ensure dry sheet metal parts. Moisture will lead to rust formation and, therefore, reduced part quality even in scrap. In the push-out test, the specimens were stored in water, as this is generally recommended to achieve best results in the strength of the UHPC [12]. This way, we test the specimens at their maximum strength. Based on the results, a storage strategy for possible UHPC forming tools should be developed.

As there is no further information about the temperature stability of the formed compounds, we performed no heat treatment to speed up the curing process for any of the test specimens.

### 2.4. Sheet Metal Material

**Figure 3:**
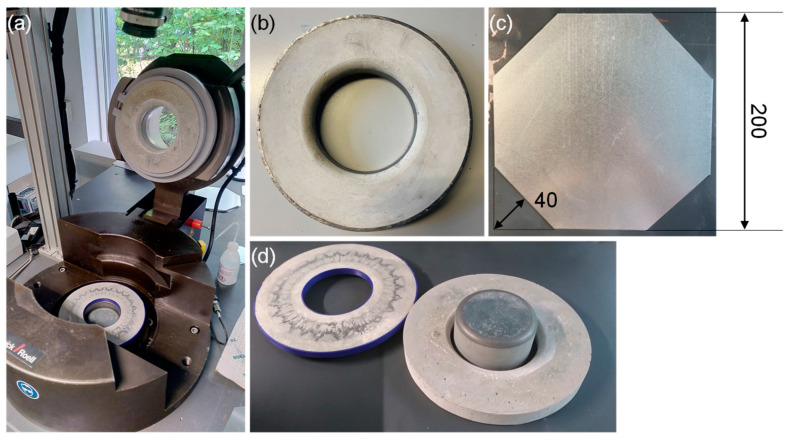
Setup for deep-drawing experiments. (**a**) UHPC-forming tools mounted on the sheet metal testing machine, (**b**) deep-drawing die made of UHPC in a restrained condition, (**c**) initial blank geometry, and (**d**) deep-drawing blankholder, punch, and die made of UHPC.

Sheet metal material 1.0963 following DIN EN 10346 [37] with a thickness of 1 mm was used. The material is a soft, hot-dip galvanized steel for cold forming. The initial blank geometry, shown in Figure 3c, is a square geometry with a side length of 200 mm. The corners are cut by 40 mm at a 45° angle.

## 3. Methods

### 3.1. Test Setup of the Free Volume Increase Test

The density of the free volume increase specimen is determined by Archimedes’ principle. The method is based on weighing a dry sample in air and fully immersing it in water. The density is determined on the assumption that the buoyancy force is equal to the amount of liquid displaced by the body. We determined the density of all specimens after 24 h and after one week.

### 3.2. Test Setup of the Push-Out Test

The push-out test used here follows the setup and execution of the standardized procedure for adhesives in DIN EN ISO 10123 [38] for the determination of shear strength. Here, the testing happens directly in the connection area. In the research field of UHPC, push-out tests are mostly used to investigate the direct shear of the interface of rebar with UHPC (e.g., Ref. [39]) or connectors embedded in UHPC (e.g., Ref. [40]).

The push-out test setup consists of a punch, a downholder with guide bushing, and a die. A schematic of the push-out test setup with notable dimensions in mm is given in Figure 2c. The punch has rounded edges with a radius of 50 µm. The clearance between the punch and die runs 3.75 mm all around. The punch force F_Punch_ acts centrally on the UHPC part of the specimen far from the steel ring. It is not the adhesion between the UHPC and the steel ring that is to be tested but the strength of the UHPC itself. The downholder screwed on the die keeps the specimen centered in place. The die also has rounded edges. The push-out test setup is mounted in the universal testing machine AllRound Line Z 150 by ZwickRoell GmbH & Co. KG, Ulm, Germany, as seen in Figure 2a. During testing, the punch moves down with 1 mm/min until fracture occurs. The universal testing machine records the corresponding force–displacement curves with a rate of 100 Hz giving a value approximately every 1.6 × 10^−3^ mm.

### 3.3. Test Setup of the Deep-Drawing Experiments

**Figure 4:**
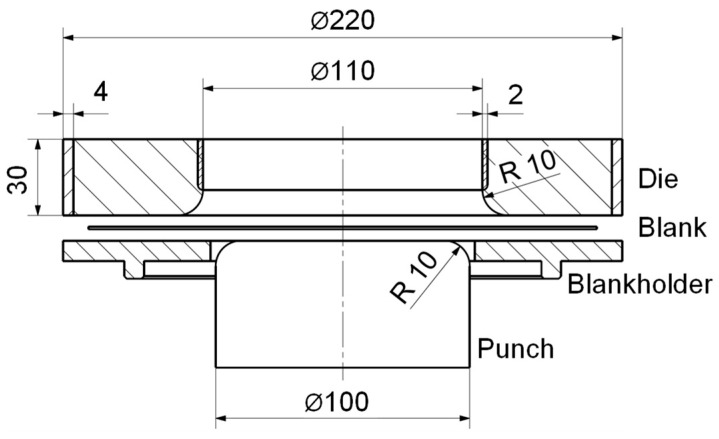
Schematic of the deep-drawing tools with notable dimensions in mm.

For the drawing experiments, the hydraulic Sheet Metal Testing Machine BUP 1000 by ZwickRoell GmbH & Co. KG, Ulm, Germany, is used. The experimental setup of the deep-drawing experiments is shown in Figure 3a. The blankholder is positioned by inserting it into a groove at the bottom part of the testing machine. The punch is centered on the hydraulic cylinder of the BUP 100 and secured against rotation. The sheet metal and die are centered and directly placed on the blankholder. The blankholder and punch are made of UHPC fabricated with the base composition in Table 1 and depicted in Figure 3d. The PLA cast of the blankholder stays during the experiments to allow for more plastic deformation and reduce the risk of breaking. The punch has a diameter of 100 mm with a 10 mm forming radius. The diameter of the open die is 110 mm, and the outer diameter is 220 mm. The die has a height of 30 mm. Those dimensions are equal for the die made of expansive UHPC in a restrained condition (Figure 3b) and the reference die made from UHPC (Figure 3d). If the die is attached to the head of the tool body, as in this case, it is subjected to a significantly more complex state of stress during forming. The punch and blankholder are mainly subjected to compressive stress, which is advantageous for UHPC. For this reason, only the die is tested in two ways. The dies are used in drawing experiments until failure. A schematic of the deep-drawing tools with notable dimensions is given in Figure 4.

The parameters of clamping force and drawing depth of the deep-drawing experiments were set before testing. First, the sheet metal is clamped between the blankholder and die with the clamping force F_C_. The punch then moves upward with a velocity of 1 mm/s and forms the cup. The clamping force was set so no flange wrinkling occurs in the formed cup. The forming force F_F_ is measured under the punch by oil pressure. The resulting punch force and actual drawing depth of the cup are measured after the experiments by the testing machine. The sample rate of 100 Hz gives a value of approximately every 0.01 mm of the punch travel. The data are given in Table 4 for the reference die made of UHPC and in Table 5 for the deep-drawing die made of expansive UHPC in a restrained condition.

## 4. Results and Discussion

### 4.1. Results of the Free Volume Increase Test

**Figure 5:**
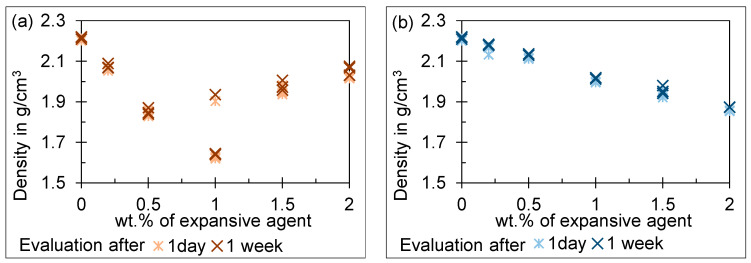
Change in the density by free volume increase of the specimen for (**a**) expansive agent 1 and (**b**) expansive agent 2, measured after one day and one week stored dry at room temperature.

The results for the density of the specimens with expansive agent 1 in different additions are given in Figure 2a and in Figure 5b for expansive agent 2.

**Figure 6:**
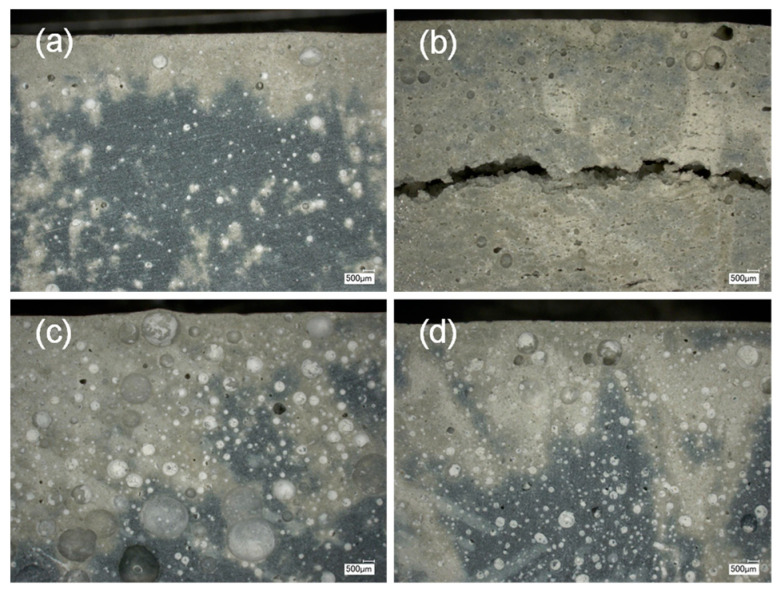
Optical microscope images with a 20× magnification of UHPC specimen after free volume increase with (**a**) no addition of an expansive agent, (**b**) 1 wt.% expansive agent 1, (**c**) 2 wt.% expansive agent 2, and (**d**) 1 wt.% expansive agent 2.

The results for specimens mixed with expansive agent 1 show no clear trend after one day and one week. With an increasing amount of expansive agent 1, the density first drops after it increases again at 1 wt.%. It should be added that the scatter is particularly high at 1 wt.%. An explanation for this can be based on the optical microscope images of the specimen. Figure 6b shows a section through a specimen with 1 wt.% expansive agent 1 in 20× magnification. A horizontal crack appears to run through the specimen. Therefore, we cannot rule out that water enters and thus falsifies the density determination. The results for specimens mixed with expansive agent 2 show a clear reduction of density with an increasing amount of expansive agent measured after one day and one week. These results are consistent with the microscope images. The pore volume seems to be increased for 1 wt.% (Figure 6d) and even more at 2 wt.% (Figure 6c) of expansive agent 2 compared to UHPC without additives (Figure 6a). Additionally, the decrease in density after one week compared to the results after one day is a result of drying.

### 4.2. Discussion of the Free Volume Increase Test

The results for specimens mixed with expansive agent 1 show an inconsistent trend. With an increasing amount of expansive agent 1 from 0.2 to 1 wt.%, the density drops. This corresponds with the expected behavior. If more than 1 wt.% is added, the density starts to stabilize around 2.0 g/cm^3^. At 1 wt.% of expansive agent 1, scatter is exceptionally high with a coefficient of variation of 8.56%. We expect the expansion behavior introduced by agent 1 into the UHPC to be the reason for the scatter as well as the inconsistent trend. Expansive agent 1 leads to cracks in the UHPC matrix (see Figure 6b) when high amounts are added. If water enters through the cracks, the volume measurements are falsified, as they are based on weighing the specimens in air and in water. The cavities fill with water during weighting and the buoyancy force is reduced compared to specimens with closed cavities of equal size. This can be observed by a smaller weight measured in water, whereas the weight of the specimen measured in air is nearly constant. This leads to the nearly constant density observed for high amounts of expansive agent 1. It additionally explains the high scatter at 1 wt.% of expansive agent 1. Either the density drops linearly as expected from the amounts added before or as water enters the specimen, falsifying the result.

Based on the free volume increase tests and the microscope images, expansive agent 2 appears to be suitable for making UHPC-forming tools. Unlike agent 1, the UHPC matrix does not crack. This potentially introduces a weak point in the UHPC-forming tools. The increasing gas volume with an increasing amount of aluminum-based expansive agent is consistent with the state of the art [25,26]. We expect the pre-straining force to increase with the restrained volume. Therefore, a higher expansion volume, meaning a smaller density of the expansive UHPC, is preferred.

### 4.3. Results of the Push-Out Test

**Figure 7:**
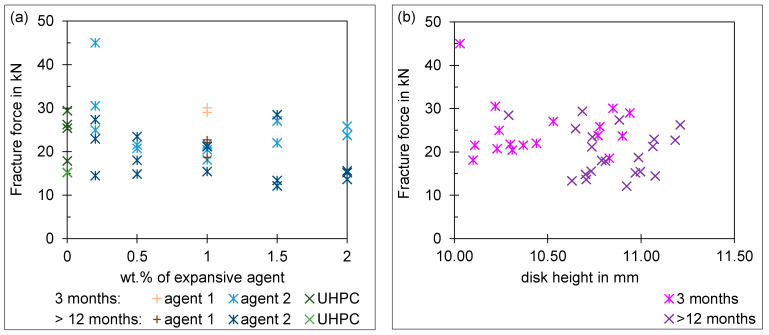
(**a**) Fracture force for specimens aged 3 months and more than 12 months; (**b**) fracture force versus push-out specimen’s height.

The fracture force of the tested push-out specimen is given in Figure 7a. The fracture force varies between 15 and 45 kN, while the punch travels between 0.3 and 0.6 mm before fracture. The height of the push-out specimen has no influence on the fracture force, as shown in Figure 7b. The same applies to the punch travel up to fracture; a later break does not result in a higher bearable force on the specimen. In addition, there is no major difference between UHPC specimens with or without an expansive agent. A higher addition of expansive agent 2 does not result in a higher fracture force. These trends are valid for specimens tested after 3 and 12 months. An aging effect of the specimens can be recognized. The fracture strength is lower after 12 months than after 3 months. However, the adhesion of the UHPC mixture in the steel ring improves in specimens made of expansive UHPC in a restrained condition. In these specimens, the cohesion of the steel ring and UHPC matrix remains intact even after fracture.

### 4.4. Discussion of the Push-Out Test

The results of the push-out test show a high scatter for all specimens regardless of the UHPC mixture. The reason for this may be the manual mixing of the UHPC mixture in combination with the small quantities added. In addition, there is no major difference between specimens with or without expansive agent, even if different amounts of expansive agent 2 are added. The same has been observed in other studies. Initially, aluminum powder increased the volume and length of cement samples, and then drying shrinkage overcame and reduced the strain such that after 120 days (approx. 4 months), the amount of strain reached zero [41]. Another influence is the hand grinding of the specimens on both sides. However, this step cannot be omitted with regard to the use of the concept for forming tools. Forming tools are always integrated into a tool body, which is necessary for mounting in a forming press. Another reason could be that the steel ring itself carries a lot of load. The effect achieved by the pre-stressing of the UHPC is, therefore, not measurable. The effect of the pre-stressing is counteracted by the decrease in the effective cross-section due to an increased pore volume. Depending on how the pores are distributed in the test specimen, they weaken the specimen to a greater or lesser extent. This leads to high scatter in the results.

Based on the principle idea of high pre-stress due to volume increase and the consistency of results of the push-out test, UHPC with 2 wt.% of expansive agent 2 is used for the forming tests. Specimens made of restrained UHPC with 2 wt.% of expansive agent 2 had high fracture forces with low scatter. Another reason for this is the improved adhesion of expansive UHPC in a steel ring and the hypothesis that an outer steel ring can itself absorb forces in the radial direction and thus relieve the UHPC.

### 4.5. Results of the Deep-Drawing Experiments

**Figure 8:**
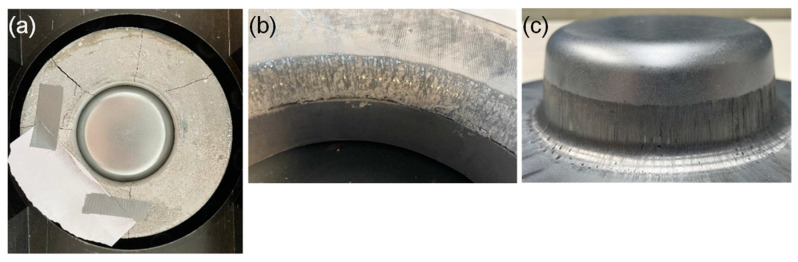
(**a**) Reference die made of UHPC, broken inside experimental setup; (**b**) deep-drawing die made of expansive UHPC in a restrained condition, after drawing experiments with degraded forming radius; (**c**) cup drawn with degraded die made of expansive UHPC in a restrained condition.

The deep-drawing die made of UHPC breaks after seven strokes at a forming force of 83.6 kN and a drawing depth of 25 mm. The die broke due to several complete cracks in a radial direction before the finish of the stroke, as shown in Figure 8a.

The deep-drawing die made of expansive UHPC in a restrained condition withstood a maximal load during deep drawing of 111.46 kN at a drawing depth of 35 mm. The drawing depth reaches a maximum of 45.04 mm limited by the testing machine. The failure mode, compared to the die made from UHPC only, changed to the surface degradation of the drawing radius, as seen in Figure 8b. The degradation after 15 strokes can also be seen in the reduced surface quality of the cup surface by marks in the zinc coating of the material, as depicted in Figure 8c.

### 4.6. Discussion of the Deep-Drawing Experiments

**Figure 9:**
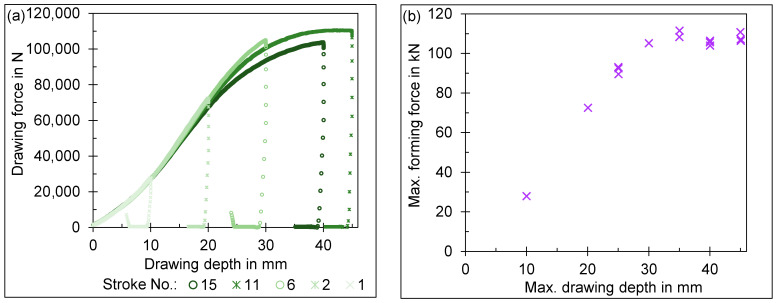
(**a**) Force–displacement curves for drawing experiments, (**b**) maximum forming force over the maximum drawing depth.

In the drawing experiments with the die made of expansive UHPC in a restrained condition, the maximum forming force increases with the drawing depth until the drawing depth reaches 35 mm. Above a drawing depth of 40 mm, the maximum forming force no longer increases with the drawing depth. This is clearly shown in Figure 9b. This can be attributed to the size of the sheet metal. With increasing drawing depth, the flange on which the clamping force acts becomes significantly smaller. The sheet now slides more easily into the drawing clearance and the drawing force no longer increases with the drawing depth from this point onwards. This can also be seen in the force–displacement curves in Figure 9a. The force–displacement curves also show that later drawing tests (shown in a darker shade of green) are shifted downwards compared to earlier drawing tests (a lighter shade of green). One reason for this is the increasing zinc layer on the drawing radius, which reduces the friction between the UHPC die and the sheet and thus the needed forming force [42,43,44]. On the other hand, the degradation of the drawing radius should be mentioned here. With increasing drawing radius, the forming force required for drawing the cup is reduced [45,46,47]. With increasing degradation of the drawing radius, the part quality and dimensional accuracy decrease, limiting the use of UHPC tools in industrial applications. More information about the wear of UHPC tools can be found in previous works [48].

## 5. Conclusions

An aluminum powder-based expansive agent was chosen based on the free volume expansion it induced in UHPC made of a ready-to-use binder. In push-out tests, the amount of pre-stressing induced by constraining said mixture with an outer steel ring was examined. With deep-drawing experiments, UHPC dies made of expansive UHPC in a restrained condition were tested versus UHPC dies without reinforcement.

In this work, we aimed for the usage of expansive UHPC in a restrained condition for deep-drawing tools. This limits the results of this study. The effects of the storage conditions and duration, wet or dry, in the cast or demolded, on the free volume expansion, as well as the effect on pre-stress, are not answered satisfactorily. To ensure that the maximum effect of pre-stress is measured, the push-out test should be repeated right after the demolding of the specimen. The literature and this study show that the effect of the restrained expansion on the strength of UHPC decreases with time. Additionally, it should be clarified to which extent the steel ring itself supports the push-out test specimen. Push-out tests of the UHPC specimen without a steel ring could help to achieve this. The molds used in this work are not conventional molds used for casting UHPC or concrete structures. The molds themselves induce an influence that has not been investigated further. PLA is elastic and the manufacturing process of fused filament fabrication is not precise. In this work, the flexibility of the shape of the UHPC part was key to the production of the forming dies and could not be replaced by conventional molds.

The following conclusions can be drawn from this work:

(1) Aluminum powder-based expansive agents lead to an increase in volume (or decrease in density) of UHPC by increasing the gas content.
(2) The expansive agents used did not induce the same expansive behavior in UHPC even though both are based on aluminum powder. The careful testing of commercial products is advised.
(3) After 3 and 12 months, no notable pre-stressing with aluminum powder-based expansive agents can be achieved when restraining the expansion of the UHPC.
(4) Reinforcing a deep-drawing die with an inner and outer steel ring improves performance compared to a UHPC die without reinforcement.

Future research to prepare industrial applications should explore the possibility of using the concept of pre-stressed UHPC in a restrained condition with non-rotationally symmetric tools. Rotationally symmetric tools offer the advantage of symmetric pre-stress around the whole tool. Moreover, a steel tube can be used and no preform has to be manufactured specifically for every geometry part. It has to be investigated to determine if the concept is applicable to closed dies, as is standard in industrial applications. Here, only an outer steel ring can be used to mechanically restrain the expansive UHPC. Since the effect of the aluminum powder-based expanding agent was not sufficient, comparative experimental data on different expanding agents under the same test conditions should be sought.

## Figures and Tables

**Table 1:** Base composition of UHPC given in added mass per volume.

Binder Nanodur Compound 5941	Water	Superplasticizer ADVA Flow375 (BV/FM)
kg/m^3^	kg/m^3^	kg/m^3^
2100.00	316.00	30.00

**Table 2:** Water–binder ratio (w/b) of specimen according to the wt.% of expansive agent.

**wt.% of Expansive Agent**	0.2	0.5	1	1.5	2
**w/b**	0.1647	0.1644	0.1631	0.1623	0.1615

**Table 3:** Composition of UHPC used for deep-drawing experiments.

	Total Volume	Binder	Water	Super- Plasticizer	Expansive Agent 2
	cm^3^	g	g	g	g
Restrained expansive UHPC	850	1785.00	268.60	25.50	35.70
UHPC	850	1785.00	268.60	25.50	0.00

**Table 4:** Parameters and results of deep-drawing experiments with reference die made of UHPC.

No.	Clamping Force F_C_	Set Drawing Depth	Max. Forming Force F_F_	Drawing Depth at Max. f_f_	Measured Drawing Depth
	kN	mm	kN	mm	mm
1	10	10	25.30	10.01	10.06
2	10	10	25.25	9.97	10.01
3	10	10	25.42	9.94	9.98
4	10	20	68.26	19.94	19.99
5	10	20	65.78	19.94	20.00
6	10	20	64.58	19.94	20.00
7	10	25	83.62	24.99	25.06

**Table 5:** Parameters and results of deep-drawing experiments with die made of expansive UHPC in a restrained condition.

No.	Clamping Force F_C_	SET Drawing Depth	Max. Forming Force F_F_	Drawing Depth at Max. F_F_	Measured Drawing Depth
	kN	mm	kN	mm	mm
1	10	10	27.87	9.98	10.03
2	10	20	72.49	19.99	20.04
3	10	25	89.43	25.01	25.06
4	16	25	92.42	24.98	25.04
5	20	25	93.09	21.34	25.03
6	20	30	105.08	29.95	30.01
7	20	35	111.46	34.96	35.03
8	20	40	106.43	39.96	40.02
9	20	45	106.26	42.15	45.04
10	20	45	107.04	43.26	45.04
11	20	45	110.70	42.15	45.00
12	20	35	111.46	34.96	35.03
13	20	35	108.24	34.94	35.00
14	20	40	105.56	39.89	40.00
15	20	40	103.92	39.97	40.03

## Data Availability

The raw data supporting the conclusions of this article will be made available by the authors on request.
